# Adolescent idiopathic scoliosis and back pain

**DOI:** 10.1186/s13013-016-0086-7

**Published:** 2016-09-09

**Authors:** Federico Balagué, Ferran Pellisé

**Affiliations:** 1 Department of Rheumatology, HFR Fribourg-Hôpital Cantonal, 1708 Fribourg, Switzerland; 2 University of Geneva, Geneva, Switzerland; 3 Department of Orthopedics, NYU, New York, USA; 4 Spine Unit, Hospital Vall Hebron, 08035 Barcelona, Spain; 5 Spine Unit Hospital Quirón, 08023 Barcelona, Spain

**Keywords:** Adolescent idiopathic scoliosis, Back pain, Conservative management, Surgical treatment, Natural history

## Abstract

This broad narrative review addresses the relationship between adolescent idiopathic scoliosis (AIS) and back pain. AIS can be responsible for low back pain, particularly major cases. However, a linear relationship between back pain and the magnitude of the deformity cannot be expected for any individual patient. A large number of juvenile patients can remain pain-free. The long-term prognosis is rather benign for many cases and thus a tailored approach to the individual patient seems mandatory. The level of evidence available does not allow stringent recommendations for any of the disorders included in this review.

## Background

Scoliosis is a frequent pathology in adolescents. Young patients and their families frequently blame (minor) deformities, considered to be the cause of back pain. In our opinion, the relationship between the two is not so clear based on scientific evidence. For this reason, we here review this topic in depth.

A high prevalence of low back pain (LBP) among children and adolescents has been identified in the last few decades. A recent systematic review and meta-analysis [1] has reported a figure of 39.9 % (95 % CI ranging from 34.2 to 45.9 %) for lifetime prevalence. In terms of aetiologies, a retrospective study of almost 2000 patients less than 21 years-old referred for a spine evaluation reported that when a pathology is identified, the most frequent diagnosis was scoliosis (1439/1953), followed by Scheuermann’s kyphosis (163/1953) and spondylolisthesis (154/1953) [2]. Other series of cases have also shown similar findings [3, 4]. However, the role of spinal deformities in LBP in the general population is not clear. For this reason, we here review the recent literature on the associations between pain and the most common adolescent spinal deformity, which is adolescent idiopathic scoliosis.

Trying to establish a causal relationship between deformities and back pain is not an easy task. Since Sir Austin Bradford Hill [5] published his important paper in 1965, the difficulties and limitations in drawing conclusions on the causal relationship between two variables have been the focus of several publications [6–9].

Our aim is to review the literature to evaluate the association in adolescents between back pain and idiopathic scoliosis, reported in studies done in different settings and/or with different perspectives.

## Methodology

This paper is the product of the collaboration between 2 clinicians: an orthopaedic surgeon highly focused on deformities (FP) and a rheumatologist with a special interest in juvenile spinal disorders (FB). We have tried to find clinically relevant information that might answer some of the questions the practitioners have. Therefore this article does not aim to be a systematic review as we have not applied strict methodology as recommended, for example, by the Cochrane collaboration, for such reviews. We began with a bibliography search limited to MEDLINE and expanded this body of literature with a search of the publications cited in the selected articles. The search was done using several key words for back pain (backache OR back pain OR low back pain OR lumbar pain OR vertebral pain OR spinal pain) as well as for the age category of interest (adolescent OR teenager OR juvenile OR paediatric OR infant OR children). We then limited the search to meta-analyses and expanded it to include “systematic reviews”, and “cohort or longitudinal studies”, if the information gathered was too limited with the most stringent criteria. Our focus was on back pain and consequently the articles not including data on symptoms were excluded except for the Epidemiology section.

## Adolescent idiopathic scoliosis (AIS) and back pain

According to the glossary of the Scoliosis Research Society (SRS) (http://www.srs.org/professionals/online-education-and-resources/glossary/revised-glossary-of-terms), idiopathic scoliosis can be defined radiographically as a lateral curvature of the spine greater than or equal to 10° Cobb with rotation, of unknown aetiology.

The possible relationship between scoliosis and back pain can be approached from different angles. Some examples may be:

- comparing the epidemiological data of the two diagnostic entities;
- examining the prevalence of back pain in adolescents with scoliosis or the prevalence of scoliosis among back pain sufferers;
- examining the prevalence of back pain over time in scoliosis left untreated; or
- examining the impact of treatment of the deformity on back pain.

### Epidemiology

#### Prevalence of scoliosis

Adolescent idiopathic scoliosis is considered a quite common disorder, with a pooled referral rate for radiography of 5.0 %, according to a meta-analysis of 36 studies looking at the effectiveness of school scoliosis screening [10]. According to the definition used, the overall prevalence of AIS has been reported to range between 0.47 and 5.2 % in a recent review [11]. There are cases of scoliosis secondary to other pathologies, but those idiopathic (i.e. AIS) are by far the most frequent. In the series published by Rogala et al. [12], all but 9 of 1231 cases of structural scoliosis were idiopathic. We have limited this section of the review to adolescents or young adults with AIS.

There are other studies that have prospectively evaluated the epidemiology of scoliosis and its natural history in large samples of adolescents, but the most frequently referenced do not include any information on symptoms [11–15]. Even the recent meta-analysis previously mentioned did not report data on back pain [10].

Scoliosis can be associated with other deviations from the normal spinal morphology. For example, in the series reported by Deacon et al. [16], 35 out of 50 adolescents were diagnosed with both Scheuermann’s disease and scoliotic curves. Overall, 43 curves were identified in these patients, which were divided in 2 types: 13 were apical at the same level as the kyphosis, and 30 occurred in regions of compensatory lordosis. Finally, in the small series reported by Blumenthal et al. [17], 4 out of 13 cases of lumbar Scheuermann’s also had scoliosis but none required treatment.

#### Pain associated with scoliosis

Regarding associated symptoms, a German study evaluated the data of more than 640,000 youths included in an insurance database. ICD diagnosis codes M40-43 would be relevant for the purpose of our review (M40: kyphosis and lordosis; M41: scoliosis; M42: spinal osteochondrosis; and M43: other deforming dorsopathies). For scoliosis, data from 2002 showed the following means in % (girls/boys) for prevalence at 0–14 years of age and at 15–24 years: M41: 2.31 (2.51/2.12) and 3.44 (3.80/3.07) [18]. Ramirez et al. [19] reported on more than 2400 subjects with AIS. Of these, 23 % reported back pain at the time of diagnosis. An additional 9 %, initially free of pain and managed with observation alone, developed pain during follow-up. A study from Japan including more than 30,000 adolescents concluded that the subgroup with scoliosis had an approximately 3 to 5 fold increased point and lifetime prevalence of backache in the upper and middle right part of the back [20]. In a prospective multicentre study, Lonner et al. [21] compared three groups of adolescents including 894 with AIS and 31 healthy controls. The pain scores on the specific subdomain of the SRS-22 questionnaire were 4.15 in the scoliosis group and 4.24 among the controls, which is not a significant difference.

A retrospective chart review of a random sample of 310 individuals (10–17 years) selected from all cases of AIS referred to a Canadian university hospital has been published recently [22]. The authors concluded that the prevalence of back pain was “moderately high” but the reported data on pain do not seem very homogeneous (gathered by the attending physicians, or reported by medical references, or by letters from the parents, narrative or through a pain score, with or without a specific topography of pain recorded, etc.). Of note, severe pain was documented in only 1 % of the charts [22].

A prospective multicentre study including 744 patients (621 females) surgically treated for AIS addressed the differences between genders in functional outcome. Before surgery, males were aged an average of 15.2 years and females 14.0 years (*p* < 0.001) with no significant differences in maximum Cobb angles (F: 53.3° and M: 55.9°) or Risser grades (F: 3.2 and M: 3.5) [23]. At baseline, the scores on the pain domain of the SRS-30 were 4.1 among girls and 4.3 in boys, below the statistical significance threshold [23].

A recent meta-analysis has been published comparing selective versus non-selective thoracic fusion in Lenke 1C curves [24]. Preoperative data on pain using the SRS-22 has shown slightly different scores between the two groups that is 4.13 (0.77) in the selective fusion group versus 3.92 (0.79) in those undergoing non-selective procedures. The difference is statistically significant (*p* = 0.038), but is at the limit of the minimum clinically important difference (MCID), reported to be 0.20 [21, 25, 26]. As any other tool, the questionnaires used to gather information on patients with scoliosis have some limitations. An American study evaluated SRS-22 performance in 450 healthy adolescents (mean age: 16 years; range: 9.3 to 21.8 years). Concerning specifically pain, the mean score was 4.3 ± 0.6 with males scoring a bit higher (actual figures by gender not reported but *r* = 0.103, *p* < 0.05) [27]. Moreover, ethnicity was also a significant factor with African Americans scoring significantly higher at 4.5 (i.e. less pain) than Hispanics (4.3). Other socio-demographic variables were significantly associated with different domains of the questionnaire [27].

Other studies have also shown that culture and ethnicity have an influence on outcomes with Caucasians reporting more pain than East Asians on the SRS-30 [28, 29]. Finally, in a Polish study, the living environment has also been reported to influence the results of the SRS-24 questionnaire, with rural patients reporting more pain than those from an urban environment [30].

The Pediatric Outcomes Data Collection Instrument (PODCI), a multidimensional tool developed in North America (also known as POSNA for Pediatric Orthopedic Society of North America) [31], was used to evaluate 102 patients with AIS (as well as other groups of patients with different pathologies) [32]. Scores of these patients were compared to those of a small group of 27 “healthy” adolescents evaluated in a different study [33]. Of these 102 patients, 95 (86 girls) filled in the patient questionnaire. The scores of the two groups for the comfort/pain scale were 86.7 ± 14.5 for the healthy group versus 75.2 ± 22.4 for the AIS group. Each dimension is scaled from 0 to 100, with 100 the most favourable outcome. The result is statistically significant at *p* < 0.05; however, the differences between the two groups do not seem clinically relevant when compared with the properties of the same tool described elsewhere [34].

Considering another perspective on the association between symptoms and scoliosis, a study carried out on 1743 men in the military (range: 18–30 years) found a prevalence of idiopathic scoliosis of 6.65 % among those with no symptoms and free of any lytic or olisthetic lesions [35]. Of note, none had a Cobb angle >20°. Prevalence ranged from 13.3 to 23.8 % in the symptomatic and asymptomatic subgroups with uni- or bilateral pars break and 18.3 % among those without any lesion of the posterior arch but reporting back pain.

Clark et al. [36] have just published the results of a prospective, population-based, birth cohort study with complete data on 3184 participants. Subjects were evaluated at age 15 for scoliosis using total-body dual-energy X-ray absorptiometry (DXA) and surveyed at age 18 for pain and function. A multivariable analysis shows a significant association between small spinal curves (≥6°) at age 15 and self-reported back pain at age 18. Spinal curvature is also associated with days off school and avoidance of activities.

It appears quite clear from comparing the prevalence of back pain and scoliosis that the latter cannot be the main explanation for LBP for a majority of adolescents reporting such symptoms, although scoliosis playing a role in some patients cannot be ruled out. However, comparing the results of different studies does not allow any firm conclusions to be drawn on the possible causal relationship between different variables. Nevertheless, the compelling and overwhelming predominance of girls in the adolescent cohorts with idiopathic scoliosis (up to almost 90 % in some series, such as that published by Théroux et al. [22]) casts doubt on the aetiological role of scoliosis on back pain in the general population, at least among boys. Another piece of information that suggests a limited role of scoliosis in back pain is the weak or even absent correlation between the magnitude of the curves measured by the Cobb angle and the presence of pain [22, 28, 37, 38].

The main references quoted in this section are summarized in Table 1.

**Table 1:** Summary of the main publications including data on pain presented in the order of citation in the manuscript

Reference 1st author/year	Design	Tools used for Pain	Results	Comments
N. Ramirez/JBJS 1997 [19]	Retrospective study of 2442 patients with AIS. Mean age was 14 years for those with back pain and 13 years for those who did not have pain.	History of back pain	Back pain was reported by 23 % at the time of presentation. Of 210 patients managed with observation only and who were pain-free initially, 9 % reported back pain during follow-up (about 3 years).	Pain was associated with age >15 years or Risser sign ≥2 but not with gender, type or magnitude of the curves.
T. Sato/Eur Spine J 2011 [20]	Epidemiological study including 32,083 students without scoliosis and 51 with AIS. Age range was 9–15 years.	Questionnaire Severity of back pain defined according to functional limitation (3 categories)	Adjusted OR of back pain (point or lifetime prevalence) was 2.29 in the scoliosis group compared with the controls. Pain was also more severe, had longer duration and more recurrences in the scoliosis group.	The difference was highly significant only for pain located in the right scapular area. No difference was found for lumbar pain.
B. Lonner/Spine 2013 [21]	Prospective pretreatment multicenter and retrospective chart review including 894 patients with AIS (mean age 14.9 years) who were compared with 106 patients with Scheuermann’s kyphosis (mean age 16.1 years) and with 31 healthy adolescents (mean age 14.2 years).	SRS-22	Mean Pain scores were 4.15 in the AIS group versus 4.24 among healthy controls.	MCID for Pain was set at 0.2. Patients with Scheuermann kyphosis reported significantly more pain than AIS patients
J. Theroux/Pain Res Manag 2015 [22]	Retrospective review of a random sample of 310 charts of AIS adolescents. Mean age was 13.9 years for girls and 14.5 for boys.	Documentation of back pain from different sources. Previous surgery and other spinal pathologies were exclusion criteria.	Almost half of the patients (47.3 %) had chart-documented back pain, most frequently lumbar pain. Pain intensity was specified in only 21 % of charts and described as mild in 9.4 %, moderate in 11 % and severe in 1 % of cases. Pain intensity was not correlated with the Cobb angle.	No comparison group.
D.W. Roberts/Spine 2011 [23]	Longitudinal cohort study comparing outcomes before and after surgery. *N* = 744 patients. Mean age was 15.2 years for boys and 14.0 in girls.	SRS-30	Preoperatively Pain scores were 4.1 for females and 4.3 for males. Of note, the latter were 1.2 years older than females (average age 15.2 versus 14.0 years).	Despite boys were significantly older, the baseline differences between genders were N.S.
A.J. Boniello/J Neurosurg Spine 2015 [24]	Meta-analysis of preoperative data limited to patients with Lenke type 1C curves. 1 prospective and 6 retrospective case-control studies. Overall 488 patients. Mean age for each group: 14.7 and 14.8 years (N.S.).	SRS-22	Baseline data from the largest multicenter study showed Pain scores of 4.13 ± 0.77 in the group eventually undergoing selective fusion Vs 3.92 ± 0.79 in those later fused nonselectively.	
J. Bago/Eur Spine J 2009 [25]	Study designed to identify Minimal important differences in 91 AIS patients undergoing surgical procedures. Mean age was 18.1 years (range 10–38 years).	SRS-22	Preop scores were 3.8–3.9	MID for Pain dimensions was identified at 0.6
L.Y. Carreon/Spine 2010 [26]	Longitudinal cohort (735 girls & 152 boys. Mean age 14.3 years) to evaluate MCID	SRS-22 & SRS-30	Baseline scores for Pain: 4.1 ± 0.71	MCID for Pain domain: 0.2
K. Verma/Spine 2010 [27]	Healthy adolescents. Anonymous survey *N* = 450 / 16 (10–22) years	SRS-22	Mean score for Pain domain was 4.3 ± 0.6. Males had higher score. African Americans scored higher than Hispanics.	Normative baseline in healthy adolescents
L.J. Morse/Spine 2012 [28]	Preoperative comparison of 6 ethnic groups of children with AIS. Total = 1853 composed of US white (1234), black (213), Hispanic (78), and Asian (29), as well as native Japanese (192) and Koreans (107). Overall mean age was 14.85 years ranging from 14.34 (Hispanics) to 14.97 (white). There were statistically significant differences between groups in terms of age, gender, BMI, and major curve magnitude.	SRS-30	The scores for the Pain domain ranged from 4.52 ± 0.51 among the Japanese patients, to 4.04 ± 0.72 in the US white patients (*P* < 0.001).	Whites reported more pain than Japanese and Koreans. The authors recommend taken into account cultural and ethnic differences when counseling patients.
K. Watanabe/Spine 2007 [29]	Comparison of 2 groups of 100 AIS patients each, one American and the other Japanese. Both groups were comparable with regard to age (mean age was 15.0 years in Americans and 14.9 years in Japanese), gender, curve location, Cobb angle and thoracic kyphosis.	SRS-24	Scores for the total pain domain were 3.7 ± 0.8 among Americans and 4.3 ± 0.4 in the Japanese group	The authors highlight the cultural differences and suggest that a cross-cultural comparison of the SRS-24 content is necessary.
E. Misterka/Med Sci Monit 2012 [30]	Retrospective study comparing 20 rural and 40 urban Female Polish patients with AIS with ≥ 2 years follow-up after surgery for AIS	SRS-24	Mean Pain scores were 4.4 in the urban group and 4.3 in the rural one (NS).	Some differences between groups were found but the authors did not end-up with strong conclusions based on the environment.
J.A. Lerman/Spine 2002 [32]	102 adolescents with AIS (15.3 years), 47 with congenital scoliosis and 84 “normals”.	PODCI (Pediatric Outcomes Data Collection Instrument). All dimensions scaled 0–100 where highest is best.	Comfort/Pain scores self reported by adolescents (*N* = 95) (parents’ questionnaires NOT included here) respectively were 86.7 ± 14.5 for “Normals” and 75.2 ± 22.4 in the AIS group (*P* < 0.05)	No significant differences in Comfort/pain scores according to age, Cobb angle or curve location.
R.J. Haynes/J Pediatr Orthop 2001 [33]	Only the 1st administration of the questionnaire included. Parents’ questionnaire for patients 2–18 years. Patients’ questionnaire for those aged 11–18 years.	POSNA (Pediatric Orthopaedic Society of North America)		
L.N. Pellegrino/J Spinal Disord Tech 2014 [37]	Prospective observational study pre- and postoperative of 33 patients (mean age 15.6 years).	SRS-30 and SF-36	Preop mean SRS pain score was 3.95 ± 0.09 and SF-36 61.00 ± 4.20	
P.R.P. Rushton/Spine 2013 [55]	Review and statistical analysis of the literature. Data on pain were available from 21 cohorts from 15 published studies.	SRS-22r	In 17 cohorts AIS patients reported statistically more pain than controls, in 3 cohorts patients and controls scored similarly, and in 1 study patients had less pain. However, in only 1 study was the difference clinically relevant (>MCID)	
E.M. Clark/Spine 2016 [36]	Prospective, population-based, birth cohort study. Subjects with spinal curve ≥ 6° at the age 15 (*N* = 202/3184) were surveyed for back pain at age 18	1^ary^ outcome: Pain (≥1 day in the prev. month).	At age 15 202 subjects had spinal curves ≥6°. Median curve size 11°. Curves ≥25° were found in 11 participants.	Spinal curves were identified using the DXA scoliosis method.
		2^ary^ outcomes: Self-reported days off activities	Back pain was reported by 21.3 % of the subjects with curves versus 16 % of those without.	
			≥7 days off activities in the last 6 months were reported by 21.7 % of those with spinal curves versus 12.3 % of the controls.	

### Effect of treatments on back pain

In this section, the effects of different treatments on the magnitude of scoliotic curves have not been included. Such an analysis, as well as the impact of treatment in cosmetic, psychological or other issues, would be beyond the scope of this review. Further, long-term follow-up studies in middle-age adults have also not been included, as the role of degenerative changes cannot be identified.

#### Conservative management

Numerous conservative techniques have been used to treat AIS, such as acupuncture, braces, electrical stimulation, exercises, foot orthosis, osteopathy, and yoga. The most recent systematic review on braces for AIS reported long-term stability in terms of back pain (very low quality evidence) in addition to a similar outcome for quality of life and psychological and cosmetic issues [39]. The authors highlighted that performing a meta-analysis was not possible due to differences among the studies.

In a systematic review by Maruyama et al. [40], bracing was compared to observation, other conservative treatments, and surgery. Considering the effect on QoL, the evidence was considered “conflicting” for each of the 3 comparisons. As such, the authors prudently concluded only that braces “may not have a negative impact on patients’ QOL”.

The authors of the most recent Cochrane review comparing surgical and conservative management of adolescent idiopathic scoliosis did not find any papers that would allow drawing conclusions regarding back pain [41]. Several other recent review articles have not specifically included information on back pain [42–46].

Osteopathic manipulative treatments in children were reviewed by Posadzki et al. [45]. Only one study of the included references concerned the treatment of scoliosis. There is currently no evidence to support such an approach for the treatment of AIS.

In his recent review paper, Kim [47] found no evidence available for yoga, Pilates, foot orthosis or acupuncture. On the contrary, he found level I and II evidence for bracing and level II evidence in favour of scoliosis specific exercises (despite some concerns such as the difficulty of attributing all the benefit to exercises due to frequent associated bracing, the order of magnitude of the correction and some doubts concerning the level of evidence of the studies) and against electrical stimulation. Indeed, only one well-designed study published in 1995 is specifically quoted in Kim’s review. In their prospective controlled trial Nachemson et al. [48] compared 3 groups: 1 braced, 1 treated by electrical stimulation, 1 observed. The success rates were 74 % in braced, 33 % in electrical stimulation and 34 % in observed. Thus the conclusion was that electrical stimulation is comparable to natural history. Kim [47] highlights the fact that the concept of correcting scoliosis by stimulating to muscles has almost disappeared but animal studies are being done to evaluate the possibilities to influence asymmetric vertebral growth by means of electrical stimulation. Only one study cited by this review addressed the impact on pain, but this study assessed exclusively non-structural scoliosis with a mean Cobb angle < 8° [49].

A recent systematic review on the effects of exercise in patients with AIS concluded that exercises improve quality of life [50]. The data on pain is clearly reported in only two of the nine studies included in the review, of which both used the SR-22. In the RCT by Monticone et al. [51], at baseline, the scores in the pain dimension were 3.8 (0.4) in the exercise group and 3.9 (0.5) among the controls. At 1-year follow-up, the same values were 4.7 (0.2) and 4.2 (0.4), respectively. In the retrospective study by Noh et al. [52] (method of randomisation not clearly mentioned if any), the same scores were 4.5 (2.4) in the corrective spinal technique (combining the Schroth concepts and core stabilization techniques) and 3.8 (1.6) in the control group (conventional exercise) at the beginning of the trial and 4.9 (1) and 4.6 (2.4), respectively, at 3 months. Notably, both groups began with 16 patients each, but at follow-up, data were reported for only 8 subjects in the experimental group and 4 patients in the control group.

A recent trial compared an 8-week program of weekly-supervised spinal stabilisation exercises to unsupervised exercises [53]. Pain, evaluated by numerical rating scale, scored 5.4 on average in each group. After 8 weeks, the improvement was 3.9 (1.8) in the supervised group versus 2.2 (2.0) in the unsupervised group. The study has clear limitations, such as the small numbers and the dropout rate in the unsupervised group, as highlighted by the authors.

The beneficial effects of bracing in terms of pain are influenced by compliance with the SOSORT Brace Treatment Management Guidelines (SBTMG) [54].

#### Surgical treatment

A recent review of the literature with statistical analysis included 16 cohorts with data on pain. Of these, 81 % reported a statistically significant postoperative (2 years) improvement in pain, however, in only one study were the improvements clinically important [55]. Similarly, another systematic review concluded in favour of surgery to reduce the magnitude of spinal curves but stated that evidence supporting the correlation of this result with reduced pain is lacking [56].

A multicentre, prospective, consecutive clinical series reported on the prevalence and predictors of pain in AIS treated surgically [57]. Preoperative data from 1433 patients (80.4 % girls; mean curve: 56.7°) and changes in pain based on a subset of 295 cases with complete data and 2 years follow-up were reported. Pain was evaluated using the SRS-22 questionnaire. Preoperatively, 77.9 % of patients reported some back pain, mainly mild to severe. The prevalence of mild to severe pain in the past 6 months improved with surgery from 78.3 % of the subset (*N* = 295) preoperatively, to 68.8 % at 1 year and to 64.4 % at 2 years. Indeed, at follow-up, 40 % of the 295 patients reported a decrease in pain, 38.6 % no change and another 20.3 % reported an increase in pain. Of note, while reported pain decreased with treatment, analgesic use remained unchanged. Reduction in pain and absolute pain at 2 years were both correlated with patients’ perception of deformity, evaluated by means of the SAQ (Spinal Appearance Questionnaire). Such an association might be interpreted as decreasing the likelihood of a major biomechanical role of the deformity to explain pain.

The previously cited multicentre study that included 744 cases treated surgically reported outcomes at 2-years follow-up [23]. The SRS-30 pain scores improved to 4.3 in females and 4.5 among males, which was a significant difference (*p* = 0.003). Of note, although in absolute values the differences between genders were the same pre- and post-operatively (i.e. 0.2), the pre-operative difference was not statistically significantly [23].

Akazawa et al. [58] reported the results of a survey on health-related quality of life and LBP in a group of 80 patients with AIS (mean age at follow-up: 47.4 ± 6.8 years) and a matched control group (mean age: 46.7 ± 6.3 years). There was no statistical difference in terms of pain (4.3 ± 0.6 in the idiopathic scoliosis group versus 4.2 ± 0.5 in the controls on the SRS-22 pain score) and a small difference of 1 point on the Roland & Morris questionnaire (2.4 ± 4.1 in the scoliosis group versus 1.4 ± 3.1 in the control group), which is below the MCID [59].

A prospective registry based study (*N* = 584 patients) recently reported on the prevalence of postoperative pain and its relationship with preoperative pain [60]. Patients reporting postoperative pain had significantly worse preoperative pain scores (SRS-22) with a mean of 3.8 ± 0.8 versus 4.2 ± 0.7, which is both statistically (*p* < 0.001) and clinically significant.

Another multi-centred registry study of AIS patients treated surgically (*n* = 190 with 2-year follow-up and *n* = 77 with 5 years follow-up) reported on pain prevalence and trajectories after spinal fusion. Patients were evaluated by means of the SRS-30. Moderate to severe pain in the past month was reported by 35 % of patients preoperatively. The same figure was 11 % at 1-year follow-up and 15 % at 2- and 5-year follow-up. Curiously, medication usage did not change significantly: daily opioid use was reported by 1 % across all time points and weekly or less frequent use of non-opioid medication also remained stable (23 % preoperatively, 25 % at 2-year follow-up and 25 % at 5-year follow-up) [61].

A longitudinal cohort of 745 patients with AIS treated surgically was followed for 2 years and the results of the pre- and postoperative SRS-22 compared [62]. The pre- and postoperative pain scores were 4.16 ± 0.71 and 4.31 ± 0.72, respectively. The difference is statistically significant (*P* < 0.0001) but below the MCID. At 2 years, the correlation of the reported pain with the satisfaction score was 0.260, slightly lower than for appearance (0.280) and for the total SRS-22 score (0.398) but higher than for activity (0.172) or mental (0.202) scores.

We have identified two further studies comparing the effects of bracing and surgery for the treatment of AIS. A short-term study using the SRS-22 questionnaire evaluated the outcome at age 16.3 ± 1.6 years of 109 Dutch adolescents with AIS treated either by brace, surgery, or brace followed by surgery [63]. The group treated by brace only reported significantly less pain at follow-up than the two groups that received surgery (4.5 vs 4.1). Moreover, there was no interaction between pain and the time passed since the end of treatment (i.e. interval at which the questionnaire was completed) for any of the groups [63]. A Danish group had reported on the outcome at 10 years of 181 patients with AIS treated either by brace or surgery [64]. Pain was assessed by means of 6 questions using VAS. The intensity of back and leg pain was mild, with values of 2.5 (0–10 scale) for the most severe back pain within the last 2 weeks for the braced patients and 2.1 in those operated. The scores of the SF-36 were compared with a Danish control cohort. The bodily pain scores were 74.6 (95 % CI.: 68.7–80.5) in the braced subjects, and 71.4 (66.1–76.8) in the surgical cohort, which is not a significant difference. However, both values were significantly below the scores for the reference population (79.8 (77.6–82.0)) [64]. The authors of the Ste-Justine cohort did not find any major difference in self-reported pain based on preoperative characteristics, the degree of surgical correction or the distal level of fusion [65].

A recent study has shown that both the SRS-22 and SRS-24 questionnaires are able to discriminate between AIS patients with preoperative curves >80° and those <45°. Specifically concerning pain, evaluated by the SRS-22 tool, the scores were 4.2 ± 0.7 for small curves versus 3.8 ± 0.9 for large (*p* = 0.003). The corresponding values obtained with the SRS-24 were 3.8 ± 0.6 and 3.5 ± 0.7 (*p* = 0.002). Postoperatively, the same questionnaires did not show any significant difference in pain according to neither the percent correction (<40 % Vs ≥80 %) nor when stratifying into post-operative curves <11° or ≥29° [66].

A relationship between scoliosis and pain might be mediated by variables other than the curve itself. It has been shown that compared with asymptomatic volunteers, baseline data on lumbar stiffness evaluated by means of Lumbar Stiffness Disability Index (LSDI) correlates with pain (Pearson correlation “r” LSDI versus SRS-22 Pain: −0–749) and functional limitations (LSDI vs SRS-22 Function: −0.760) in patients with spinal deformity [67].

#### Untreated scoliosis

The literature comparing untreated AIS with normal controls was recently reviewed and statistically analysed [68]. Twenty-one cohort studies were included that used different questionnaires (SRS-24/23/22/22r/30). Of these, 81 % reported statistically worse pain than unaffected adolescents; however, in only 5 % of cohorts was the difference clinically important. In comparison, in patients’ self-image, almost three-quarters of the studies with a statistically significant difference were also clinically significant.

A few studies on the conservative management of adolescents with idiopathic scoliosis have included control groups not braced. In the series reported by Pham et al. [69], a subgroup of 32 patients (30 girls, age 12.5 ± 1.4 years) with Cobb’s angles 26.5° ± 2.4° was left non braced until the next follow-up visit at least 3 months later and compared with patients wearing a specific brace (Chênau brace) full-time or during the night (weaning period). No differences in terms of pain were found.

Another study compared 78 patients with AIS being braced with 136 patients being observed. The two groups were very similar except for the magnitude of the curves (24.6° for the observed subjects and 34.5° for those being braced, *p* < 0.0001). The authors used the parents’ forms for the Child Health Questionnaire (CHQ) and the American Academy of Orthopaedic Surgeons Pediatric Outcomes Data Collection Instrument (PODCI). National normative values were available for the CHQ but not for the second instrument. No significant differences between the two groups of patients (observed vs braced) were reported for any of the pain dimensions of the two questionnaires. Moreover, when compared with the scores of healthy children, those with AIS showed similar scores on the bodily pain domain of the CHQ [70].

The main references quoted in this section are summarized in Table 2.

**Table 2:** Summary of the main publications including data on the effect on pain of different treatments presented in the order of citation in the manuscript

Reference 1st author/year	Design	Tools used for Pain	Results	Comments
Conservative Treatment				
S Negrini/Cochrane Database Syst Rev 2015 [39]	Review including 7 studies (5 initially planned as RCTs and 2 as prospective controlled trails) with a total of 662 adolescents, comparing braces with other treatment.	PedsQL (only 1 item in one of the 4 dimensions focus specifically on pain).	Back pain did not change in the long term (very low quality evidence)	The authors highlight that it was not possible to perform a meta-analysis due to important clinical differences among studies.
T Maruyama/Physiother Theory Pract 2011 [40]	Systematic review including 20 studies: 2 controlled clinical trials and 18 case-control studies. No RCTs found.	Child Health Questionnaire (1 study) and VAS (1 study)	Compared with observation bracing does not influence back pain or HRQoL. Conflicting evidence reported for studies comparing bracing with other forms of treatment	
ME Alves de Araujo/J Bodyw Mov Ther 2012 [49]	RCT comparing Pilates-based therapy (*N* = 20) to weekly meetings with no therapeutic intervention (*N* = 11). Age ranged from 18 to 25 years	Borg CR 10	Pain decreased from 5.3(1.5) to 1.8 (1.9) (*P* = 0.0001) in the experimental group and from 4.4(2.3) to 3.8(2.7) in the control group (NS)	All patients were female students with minor non-structural dorso-lumbar scoliosis (Cobb angles 7.6(3.5) (Experimental) and 7.1(2.8) (Control) respectively.
M Monticone/Eur Spine J 2014 [51]	RCT comparing self-correction, task-oriented spinal exercises and education (*N* = 55) with traditional spinal exercises (*N* = 55). Evaluations pre-, post-treatment, and at 1-year follow-up. Mean age at baseline 12.5 and 12.4 years respectively (NS)	SRS-22	Pain scores at the 3 evaluations were: 3.8 (0.4), 4.6 (0.3), and 4.7 (0.2) in the experimental program and 3.9 (0.5), 4.3 (0.3), and 4.2 (0.4) in the control group. *P* < 0.001 for time, group, and interaction effects.	
DK Noh/J Back Musculoskelet Rehabil 2014 [52]	Comparison of a corrective spinal technique (CST) approach with a conventional exercise (CE) program. Two groups of 16 AIS patients each. Mean age 13.8 years Versus 14.9 (N.S.)	SRS-22	Pain scores improved from baseline to follow-up in both groups. Results were from 4.5 to 4.9 in CST group and from 3.8 to 4.6 in CE group.	The 2 groups were already small at baseline and the attrition rate was substantial with *N* = 8 (CST) and 4 (CE) at follow-up.
KA Zapata/Pediatr Phys Ther 2015 [53]	Randomized trial comparing 8 weeks of weekly supervised spinal stabilization exercises (*N* = 17) with 1-time treatment (*N* = 17). Patients with AIS aged 10–17 years	Numeric Pain Rating Scale (NPRS) Oswestry Patient-specific functional scale (PSFS)	NPRS scores improved from 5.4(1.5) to 1.5(1.8) in the supervised group and from 5.4(1.3) to 3.4(1.7) in controls (P ≤ 0.05 & > MCID) The reduction in OSW was similar in both groups and the improvement in PSFS was higher in the supervised group but the difference between the 2 groups < MCID in adults.	All the patients included in the trial had low back pain at baseline.
M Tavernaro/Scoliosis 2012 [54]	Cross-sectional study followed by retrospective case-control study to verify the impact of a complete rehabilitation team in adolescent patients with bracing (*N* = 28 AIS and 10 hyperkyphosis). Mean age 15.8 years	SRS-22	Pain scores were 3.93 ± 0.55 among those treated by the team and 3.54 ± 0.83 in those who were not (N.S.)	Other variables were significantly improved by the team management.
EM Bunge/Eur Spine J 2007 [63]	Cross-sectional evaluation of HRQoL of AIS patients after completing treatment. Patients had been braced (*N* = 36), treated by brace and surgery (31), or only by surgery (30). Mean age 16.3 years.	SRS-22	Mean scores for Pain domain in the 3 groups were: Brace: 4.5 (0.57) Brace & surg: 4.1 (0.90) Only surgery: 4.1 (0.71)	Pain was not significantly correlated with the time span between completing treatment and filling out the questionnaire.
Surgical Management				
PR Rushton/Spine 2013 [68]	Review of the literature and statistical analysis evaluating the effect of surgery on HRQoL of adolescents with AIS and 2 years follow-up.	SRS-24	81 % of the included cohorts reported a statistically significant improvement of pain. The reduction was above MCID only in 1/12 cohorts	
MC Hawes/Disabil Rehabil 2008 [56]	Systematic review of studies on surgery for AIS with ≥ 10 patients and followed for ≥2 years after surgery. 82 articles (5780 patients) included.	Trials’ results presented as “pain-free”: Yes, No, or Not tested/reported. Yes meant that most or all patients reported minimal or no pain and none reported severe, chronic, or increased pain post-op	The authors conclude that there is no evidence to support that the result in terms of reduced magnitude of the spinal curve is correlated with reduced pain.	65 % of articles did not include pain in their outcome. Of those who did, definitions were quite different
Z Landman/Spine 2011 [57]	Multicenter, prospective, consecutive clinical series. *N* = 1433 patients. Changes in pain assessed in 295 patients with complete data and 2 years follow-up	SRS-22 (Z-test for proportions used to analyze preop and postop differences)	Mild to severe pain within the last month in 73.2 % preop, 53.6 % at 1 year and 53.2 % at 2 years. Pain at rest in 70.5 % preop, 56.9 % at 1 year and 60 % at 2 years. Compared with preoperative data, at 2 years 40 % of patients reported a decrease in pain, 38.6 % no change, and 20.3 % an increase in pain.	A significant disagreement between preoperative pain reported by physicians (44 %) and patients (77.9 %) was found. Patients who were more satisfied with their appearance reported less pain.
DW Roberts/Spine 2011 [23]	Longitudinal multicenter cohort study to compare functional outcomes between genders before and after surgery. *N* = 744. Mean age was 15.2 years for males and 14 years for females.	SRS-30	Postop pain improved significantly in both genders from 4.1 to 4.3 in girls and from 4.3 to 4.5 in boys. The difference between genders is NS.	The pain reduction at 2 years does not seem clinically meaningful.
T Akazawa/Spine 2012 [58]	Case-control study to compare healthy subjects (*N* = 80) with idiopathic (*N* = 80) and non-idiopathic (*N* = 56) scoliosis patients and ≥ 21 years after surgery.	SRS-22 Roland-Morris (RDQ)	Pain dimension scores were 4.3(0.6) in AIS patients and 4.2(0.5) in controls (NS) RDQ scores were 2.4(4.1) and 1.4(3.1) respectively.	At long-term postoperative follow-up AIS patients had similar pain scores as healthy controls
TP Bastrom/Spine 2013 [60]	Review of a prospective multicenter database registry. *N* = 584 AIS patients treated surgically with ≥ 2 years follow-up. Age at surgery 14.7 ± 2 years	SRS-22 Self-reported pain Vs pain free (1^st^ 6 months post-op or 6–24 months post-op). The focus is on the patients with unexplained pain >6 months postoperative	The 2-year pain scores were 4.1 ± 0.7 in patients with postoperative pain and 4.5 ± 0.6 in the postop pain free group (*P* < 0.001). These 2 groups were also significantly different in terms of preoperative pain (3.8 ± 0.8 Vs 4.1 ± 0.7).	
CB Sieberg/J Pain 2013 [61]	Prospective multicenter registry examining postoperative outcomes of AIS patients. *N* = 169 at baseline, 1 and 2 years postop and *N* = 69 for 5 years follow-up.	SRS-30	Moderate to severe pain in the past month was reported preop by 35 % of patients. The figures were 11 % at 1 year, 15 % at 2 years, and 15 % at 5-years post-surgery. Pain often to very often at rest was reported by 43 % at baseline, 5 % at 1- and 2-years follow-up and 8 % at final follow-up.	Examining the evolution of pain from preoperatively to 5 years follow-up the authors describe 5 trajectories distinguishable on preop age, mental health, and self-image.
LY Carreon/Spine 2011 [62]	Longitudinal cohort of AIS patients treated surgically and evaluated preoperatively and 2 years postop. *N* = 745. Mean age 14.2 years	SRS-22 and SRS satisfaction	Mean pain domain scores improved from 4.16 ± 0.71 preop to 4.31 ± 0.72 (*P* < 0.0001) Pain and satisfaction domains were significantly correlated (Spearman 0.260)	The pain reduction does not seem clinically relevant
EM Bunge/Eur Spine J 2007 ^a^ [63]	Cross-sectional evaluation of HRQoL of AIS patients after completing treatment. Patients had been braced (*N* = 36), treated by brace and surgery (31), or only by surgery (30). Mean age 16.3 years.	SRS-22	Mean scores for Pain domain in the 3 groups were: Brace: 4.5 (0.57) Brace & surg: 4.1 (0.90) Only surgery: 4.1 (0.71)	A subset of patients’ satisfaction scores was compared with that of their surgeons. No significant differences were reported
MO Andersen/Spine 2006 [64]	Longitudinal study of consecutive AIS patients (*N* = 181) treated by brace (BT = 82) or surgery (ST = 99). Follow-up 9.7 years	VAS (6 items) SF-36	Among the 6 VAS scores only “Do you feel leg pain right now” was significantly different with 0.5 (0.2–0.9) in the BT group and 0.2 (0.0–0.3) in the ST group (*P* = 0.034). The results for the mean Bodily pain dimension of the SF-36 were 74.6 (BT) and 71.4 (ST) which are significantly lower than the mean of 79.8 of a 408 Danish control cohort.	The difference in pain does not seem clinically meaningful
B Poitras/Spine 1994 [65]	Retrospective cohort study of patients referred to a single center for AIS compared with a sample (*N* = 1755) of the general population. Among the 723 patients treated surgically, 555 completed the questionnaire. Follow-up 10–30 years.	Pain assessed by Postal questionnaire (taken from McGill Pain Questionnaire, Oswestry and Roland Morris) Controls evaluated by telephone survey.	Back pain in the past year was reported by almost ¾ of the responders. For controls the same figure was 56 %.	The authors found no correlations with several surgical technical variables evaluated (curve correction, number of vertebrae fused, distal level of fusion).
Untreated				
PR Rushton/Spine 2013 [55]	Review of the literature and statistical analysis to compare untreated adolescents with AIS with normal controls.	SRS-22r	Of the included cohorts 81 % reported statistically significant worse pain among untreated scoliotic patients but the difference was clinically important only in 5 % of cohorts.	
VM Pham/Ann Readapt Med Phys 2008 [69]	Comparison of 3 groups of patients with AIS 32 without brace, 41 treated full-time with a corset and 35 wearing the brace during the night only. The 3 groups were significantly different in age, Risser, Cobb angle, etc.	VAS Quality of Life Profile for Spine Deformities	No significant differences in the intensity of pain (VAS) were found	
OF Ugwonali/Spine J 2004 [70]	Cross-sectional questionnaire-based study. Patients with AIS were braced (*N* = 78) or observed (*N* = 136) Mean age was respectively 13.6 Versus 13.8 years (N.S.)	Child Health Questionnaire (CHQ Parental form-28) and PODCI	No differences in pain domains were found between the 2 groups despite the fact that Cobb angles were significantly bigger in the braced group. Regression analysis showed a significant association of the CHQ bodily pain dimension and age but not with gender, cobb angle or treatment.	No information gathered from the adolescents themselves is included in the study. The authors conclude that bracing does not decrease QoL

*QoL* Quality of Life, *HRQoL* Health Related Quality of Life, *PedsQl* Pediatric Quality of Life Inventory, *SRS* Scoliosis Research Society

^a^ this reference is included in both the Conservative management and Surgical treatment sections

## Conclusions

Back pain in adolescents is quite common, especially in girls. There is no doubt that some AIS patients are back pain sufferers; however, pain does not seem to be a major problem for the vast majority of adolescents with an idiopathic form of this type of deformity. Some elements from the literature suggest that the link between pain and scoliosis is not strongly linked with a biomechanical problem. In most studies, pain has not shown a strong correlation with the Cobb angle, untreated cases fare reasonably well from the perspective of back pain, there is a much greater difference between genders in the incidence of scoliosis compared with the epidemiological data on back pain, and patients’ self-perception of their image correlates with pain. All these findings argue against a major aetiological role of the idiopathic scoliotic deformity of adolescents on back pain. However, the impact of pain in adults’ scoliosis is entirely different [71, 72] and out of the scope of this review.

## Abbreviations

AIS: Adolescent idiopathic scoliosis
BP: Back pain
CHQ: Child health questionnaire
ICD: International classification of diseases
LBP: Low back pain
LSDI: Lumbar Stiffness Disability Index
MCID: Minimal clinically important difference
PODCI: Pediatric Outcomes Data Collection Instrument
POSNA: Pediatric Orthopedic Society of North America
QOL: Quality of Life
SAQ: Spinal Appearance Questionnaire
SRS: Scoliosis Research Society
VAS: Visual analogue scale
